# Heart rate variability as a marker of multiple organ dysfunction syndrome in deeply sedated, prepubescent patients: a secondary analysis

**DOI:** 10.1007/s10877-026-01417-z

**Published:** 2026-02-05

**Authors:** Anne Wojtanowski, Côme Bureau, Mathieu Jeanne, Morgan Recher, Julien De Jonckheere

**Affiliations:** 1 CIC IT 1403, CHU Lille, Lille, F-59000 France; 2 ULR 2694 METRICS, Univ. Lille, Lille, F-59000 France; 3 Service de Médecine Intensive-Réanimation, CHU Lille, Lille, F-59000 France; 4 Anesthesia and Intensive Care department, CHU Lille, Lille, F-59000 France; 5 ULR 7365 GRITA, Univ. Lille, Lille, F-59000 France; 6 Pediatric Intensive Care Unit, CHU Lille, Lille, F-59000 France

**Keywords:** Multiple organ dysfunction syndrome, Heart rate variability, Pediatric intensive care unit, Autonomic nervous system

## Abstract

Multiple organ dysfunction syndrome (MODS) is a frequent complication in critically ill patients. The objective of the present study in a pediatric intensive care unit was to determine the feasibility of using HRV to differentiate between deeply sedated patients with medium/high MODS and those with no/low MODS. This was a secondary analysis of data from the ANI EP clinical study (NCT04913038). Various HRV indices were computed in a 10-minute time window. A patient with a pediatric Sequential Organ Failure Assessment (pSOFA) score higher than 5 was classified into the medium/high MODS group. Forty-seven patients were selected for analysis. The Energy variable (equivalent to the standard deviation of normal-to-normal intervals) was significantly correlated with the pSOFA score (r²=-0.31, *p* = 0.03). HFnu values were higher in the medium/high MODS group than in the no/low MODS group (0.35 vs. 0.28, respectively; *p* = 0.019), and HFnu discriminated between the two groups of patients with an area under the receiver operating characteristic curve of 0.75 (sensitivity = 0.78, specificity = 0.74). Energy was slightly but not significantly lower in the medium/high MODS group (0.17, vs. 0.35 in the no/mild MODS group; *p* = 0.086). The results of our study in a PICU showed that HRV indices can differentiate between deeply sedated patients with MODS and those without. Further investigations are needed to confirm this finding and extend it to other populations.

## Introduction

Multiple organ dysfunction syndrome (MODS) is a frequent complication among critically ill patients. The reported incidence of MODS ranges from 28% to 88% in intensive care units (ICUs) and 21% to 37% in pediatric ICUs (PICUs) [1]. MODS is associated with an elevated mortality rate.

Several scoring systems are commonly used to monitor organ dysfunction in ICU and PICU patients. The Sequential Organ Failure Assessment (SOFA) score is the reference tool in adults and has an equivalent for use in the PICU: the pediatric SOFA (pSOFA) score [2]. The pSOFA score is age-adjusted and assesses six organ systems: respiratory, coagulation, hepatic, cardiovascular, neurologic and renal systems. The Pediatric Logistic Organ Dysfunction 2 (PELOD 2) score is also used to assess MODS in the PICU [3]; it is also age-adjusted and assesses five different organ systems: cardiovascular, neurologic, respiratory, hematologic and renal systems. In France, the Phoenix score [4] has been recommended by the health authorities (*Haute Autorité de Santé*) [5] for use in pediatric patients with sepsis. Like the pSOFA and PELOD 2 scores, the Phoenix score is age-adjusted. It assesses four organ systems: respiratory, cardiovascular, coagulation and neurological systems.

In order to compute these scores, clinicians need information on vital signs (e.g. blood pressure and PaO_2_/FiO_2_), laboratory variables (e.g. the platelet count and the serum creatinine level), and the administration of life-support drugs (e.g. norepinephrine and dobutamine). In clinical practice, however, these scores are not computed daily because they are time consuming to produce and often limited by missing data.

A new bedside monitoring tool that would continuously monitor changes in organ dysfunction (and hence the risk of death) in the PICU would be of great clinical value; it might help clinicians to rapidly and regularly evaluate a patient’s condition and treatment responses during the PICU stay.

MODS develops through the activation of the innate immune system, leading to excessive release of inflammatory mediators that can trigger inflammatory dysfunctions. Under inflammation, the patient develops several coping mechanisms involving the autonomic nervous system (ANS) via sympathetic and parasympathetic activities. The systemic inflammation induced by MODS could therefore be studied by the analysis of heart rate variability (HRV) which reflects the activity of the ANS [6].

In a recent publication, we systematically reviewed the literature on the use of HRV analysis to assess MODS patients in the ICU or the PICU [7]. We notably concluded that most of the studies in this field were not standardized in terms of ventilation, drug prescriptions and/or ICU admission conditions; these variations might constitute a source of bias for interpretation of the HRV data [8]. To eliminate this bias, we decided to study RR series recorded under standardized conditions (deep sedation, mechanical ventilation and out of any painful stimulation). Moreover, only one study was performed in a PICU. To the best of our knowledge, the ability of HRV analysis to detect MODS in sedated, mechanically ventilated PICU patients has not been assessed previously.

Hence, the primary objective of the present study was to assess the ability of several HRV indices to discriminate between sedated, mechanically ventilated PICU patients with no/mild MODS and those with moderate/severe MODS.

## Method and materials

### Ethics

All procedures were conducted in accordance with the relevant guidelines and regulations. The study data were generated during the ANI EP clinical study (“Evaluation of the validity of the ANI over 2 years of age prepubescent children ventilated and sedated in pediatric intensive care unit”), which was registered at clinicaltrials.gov (NCT04913038) and with the French National Agency for Medicines and Health Product Safety (reference: 2021-A00487-34) [9]. The study was conducted between June 2021 and November 2024. Each child’s legal representatives were free to refuse the child’s participation in the study.

The present report concerns a novel, exploratory, secondary analysis of the ANI EP study data. The study database was registered with the French National Data Protection Commission (*Commission Nationale de l’Informatique et des Libertés*, Paris, France; reference: DEC25-047).

### Procedures

The ANI EP study consisted of observing variations in ANI during a period of care (e.g. washing, tracheal suctioning, dressing changes…) in patients aged 2 to 10 years for girls and 2 to 12 years for boys. ANI recording began 10 min before the period of care and ended 10 min after the period of care [9]. The primary outcome of the ANI EP study was to evaluate the variations in ANI before during and after a painful procedure. Secondary outcome concerned the evaluation of the correlation between ANI and the behavioral pain scale used in PICU (COMFORT-B scale). ANI EP study therefore aimed to validate the use of ANI for pain measurement in prepubescent ventilated and sedated PICU patients. In ANI EP, we concluded that ANI might be a good candidate for assessing discomfort in intubated, prepubescent patients in the PICU. The present study aimed to evaluate the ability of ANI and other HRV markers to differentiate no/mild MODS patients from moderate/severe MODS patients using the data generated in the frame of the ANI EP. The ANI EP protocol was not pre-specified for this secondary analysis [9]. In the present analysis, only patients who had been recorded using the PhysioDoloris for at least 10 min before care (i.e., before any stimulation) and had a good-quality HRV signal (visual inspection) were included. The data were accessed after the end of ANI EP study. An information sheet describing the reuse of the study data was sent to the patients’ legal representatives.

The pSOFA, PELOD2 and Phoenix scores were not scored in the ANI EP study and were scored *post hoc* for this secondary analysis for the day on which the HRV signal was recorded. Missing data were considered normal, as it is stated by the scores. There is no consensus about the pSOFA score threshold. However, Luo et al. defined a threshold of 5 for moderate/severe MODS patients as it reflects dysfunction in at least 2 organs [10]. In this study, the patients were therefore classified as having “moderate/severe MODS” (defined as a pSOFA score above 5) or “no/mild MODS” (defined as a pSOFA score of 5 or less).The Paediatric Index of Mortality III (PIM III) was scored on admission to the PICU and was already scored for the ANI EP study [11].

### Signal processing

RR data exported from the PhysioDoloris^®^ monitor were analyzed *post hoc*, with the computation of several HRV variables (Table 1). Only ANI was evaluated in ANI EP. Any other HRV index presented in Table 1 was specifically computed for this secondary analysis. Before the HRV indices were computed, ectopic beats and RR series artefacts were detected and replaced via linear interpolation with the specific algorithm already used in the PhysioDoloris monitor [12, 13]. HRV indexes computation is describe in Table 1.

**Table 1:** HRV variables

*Time analysis*	
SDNN	The standard deviation of normal-to-normal RR intervals was computed over 100 RR intervals (corresponding to approximately 2 min). The SDNN corresponds to the overall variability of the autonomous nervous system’s activity.
RMSSD	The root mean square of successive differences between adjacent RR intervals was computed over 100 RR intervals (corresponding to approximately 2 min). The RMSSD corresponds to short-term variations in the parasympathetic nervous system’s activity. [14]
Energy	Energy is computed after the RR series is resampled at 8 Hz. It is computed in a 64-second window as the root of the sum of the squared differences between each sample and the mean value of the samples over the 64-sec window.
*Spectral analysis*	
LF	Low frequency was based on spectral analysis of the RR series: the standard bandwidth was defined as [0.05–0.15 Hz]. LF was computed via a wavelet transform (e.g. Daubechies 4-wavelet) on a 64-second window resampled at 8 Hz, using linear interpolation. LF reflects both sympathetic and parasympathetic modulations and is mainly influenced by baroreflex activity.
HF	High frequency was based on spectral analysis of the RR series: the standard bandwidth was defined as [0.15–0.4 Hz]. HF was computed in the same way as LF. HF reflects parasympathetic modulations only and is mainly influenced by respiratory sinus arrhythmia.
HFnu	The normalized HF content was defined as HFnu = HF/(LF + HF) and reflects parasympathetic activity.
*Graphical analysis*	
ANI	The Analgesia Nociception Index is a graphical index of the magnitude of normalized HF oscillations. The RR series were resampled at 8 Hz, centered, and normalized in 64-second windows. The signal was band-pass-filtered with a wavelet transform, in order to retain HF content only. The magnitude of these oscillations was measured as the surface area between the upper and lower envelopes. The ANI gives the relative magnitude of the HF spectral content on a 0-to-100 scale; it is therefore a measure of parasympathetic activity and is mainly influenced by respiratory sinus arrhythmia.

A mean value over the 10 min window was then computed for statistical analysis.

### Statistical analysis

Quantitative variables were expressed as the median [interquartile range], and qualitative variables were expressed as the frequency (percentage). Associations between HRV indices and the pSOFA, PELOD2 and Phoenix scores were evaluated in Spearman’s correlation test. Groups were compared using Wilcoxon’s test for quantitative variables and Fisher’s exact test for qualitative variables. Univariate analyses of HRV indices were used to determine their ability to discriminate between the two groups, according to the area under the receiver operating characteristic curve (AUROC), the sensitivity (Se) and specificity (Sp). To test the independence of HRV indices from confounding variables, we used multivariate logistic regression to adjust the results for potentially confounding variables. Confounding variables were defined as any variables out of laboratory variables which potentially impact the ANS and demonstrated significant differences between groups in our study. Continuous variables were not standardized or neither normalized before regression. Results of the model were presented as odds ratios (OR) and 95% confidence intervals (CI). The threshold for statistical significance was set to *p* < 0.05. All the analyses were performed with RStudio software (version 2024.09.1, Posit Software, PBC, Boston, MA, USA) [15].

## Results

### The study population

Of the 50 patients included in the ANI EP study, 47 were selected for the present secondary analysis. Two patients were not selected because of poor quality signal, and a third was not selected because his legal representatives could not be contacted. Of the 47 patients, 38 were classified into the no/mild MODS group (median [IQR] pSOFA score: 2 [1, 4]) and 9 were classified into the moderate/severe MODS group (pSOFA score: 8 [7, 9]) (Table 2).

The two groups differed significantly with regard to the proportion of females (78% in the moderate/severe MODS group, vs. 34% in the no/mild MODS group; *p* = 0.026). The PELOD 2 and Phoenix scores were higher in the moderate/severe MODS group than in the no/mild MODS (PELOD 2 score: 8 [7, 9], vs. 5 [3, 5], respectively; *p* = 0.024; Phoenix score: 4 [3, 6] vs. 0 [0, 1], respectively; *p* < 0.001) (Table 2). For respiratory variables, the groups differed significantly with regard to FiO_2_ and SPO_2_/FiO_2_; the moderate/severe MODS group had higher values of FiO_2_ (60 [35, 80], vs. 30 [25, 50] in the no/mild MODS group; *p* = 0.016) and lower values of SPO_2_/FiO_2_ (147 [116, 257] vs. 333 [283, 388], respectively; *p* = 0.006) (Table 2). Levels of certain laboratory markers of infection or the consequences of an infection were higher in the moderate/severe MODS group (serum CRP concentration: 197 [130, 273], vs. 25 [13, 93] in the no/lmild MODS group; *p* = 0.011; serum creatinine concentration: 5 [4, 8] vs. 3 [2, 3], respectively; *p* = 0.045) (Table 2).

The two groups did not differ significantly with regard to medication exposure, except for catecholamines; administration of the latter was significantly more frequent in the moderate/severe MODS group (78%, vs. 24% in the no/mild MODS group; *p* = 0.004) (Table 2).

**Table 2:** Characteristics of the study population, by MODS group

Characteristic	No/mild MODS, *N* = 38	Moderate/severe MODS, *N* = 9	*p*-value
Age, *years*	5 (3, 8)	8 (6, 10)	0.2
Sex, *female*	13 (34%)	7 (78%)	0.026
Weight	19 [15, 27]	20 [14, 28]	0.9
*MODS*			
PELOD2 score	5 [3, 5]	8 [4, 11]	0.024
pSOFA score	2 [1, 4]	8 [7, 9]	< 0.001
*Dysfunction in individual organ systems*			
Cardiovascular	1 [0, 1]	3 [2, 4]	0.006
Respiratory	0 [0, 1]	4 [2, 4]	0.001
Neurology	0 [0, 0]	0 [0, 0]	0.7
Renal	0 [0, 0]	0 [0, 0]	0.099
Hepatic	0 [0, 0]	0 [0, 1]	0.21
Hematology	0 [0, 0]	1 [0, 2]	0.0014
Phoenix score	0 [0, 1]	4 [3, 6]	< 0.001
Comfort B score	9 [7, 13]	9 [8, 11]	0.8
*Comfort type*			0.5
Borderline state, possible pain	2 (5%)	1 (11%)	
Comfort	13 (34%)	2 (22%)	
Over-sedated	23 (61%)	6 (67%)	
PIM III score	1.5 [0.5, 4.9]	1.6 [1.2, 3.4]	0.8
Death at 28 days	1 (3%)	1 (11%)	0.3
*Hemodynamic variables*			
MPB min	62 [57, 70]	58 [56, 65]	0.3
Heart Rate max	133 [117, 155]	138 [129, 157]	0.3
*Respiratory variables*			
Respiratory rate	22 [20, 25]	22 [19, 25]	0.5
VT max	188 [118, 251]	157 [140, 221]	0.4
VT/kg	8.38 [6.63, 10.06]	7.50 [5.82, 9.60]	0.3
FiO_2_	30 [25, 50]	60 [35, 80]	0.016
Arterial PO_2_	71 [55, 76] *n* = 12	66 [52, 79] *n* = 7	0.7
Mechanical ventilation, controlled	17 (45%)	2 (22%)	0.3
SPO_2_/FiO_2_	333 [283, 388]	147 [116, 257]	0.006
SPO_2_	94 [92, 96]	90 [88, 91]	
*Laboratory variables*			
Bicarbonate (mmHg)	25.8 [22.6, 27.4]	25.5 [21.0, 27.7]	0.7
Lactates (mmol/L)	1.30 [0.90, 1.68]	1.00 [0.90, 2.10]	> 0.9
Creatinine (mg/L)	3 [2, 3] *n* = 32	5 [4, 8] *n* = 8	0.045
Total bilirubin (mg/L)	5 [4, 12] *n* = 17	7 [5, 10]	0.6
CRP (mg/L)	25 [13, 93] *n* = 21	197 [130, 273] *n* = 6	0.011
Leukocyte count (x10^9^/L)	11.3 [8.2, 15.3] *n* = 31	11.6 [10.7, 13.5]	> 0.9
Procalcitonin	1 [0, 8] *n* = 7	11 [6, 12] *n* = 3	0.5
Arterial pH	7.35 [7.30, 7.39] *n* = 26	7.34 [7.29, 7.38] *n* = 6	0.8
PCO_2_	47 [43, 52]	60 [51, 65]	0.013
*Medication exposure*			
Catecholamine	9 (24%)	7 (78%)	0.004
Dobutamine	1 (11%)	0 (0%)	0.2
Dobutamine + norepinephrine	0 (0%)	2 (29%)	
Norepinephrine	8 (89%)	5 (71%)	
Dexmedetomidine	7 (18%)	2 (22%)	> 0.9
Albuterol	2 (5.3%)	0 (0%)	> 0.9
*Analgesia*			0.5
Yes	36 (95%)	8 (89%)	
*Analgesia type*			0.14
Morphine	22 (58%)	8 (89%)	
Remifentanil	2 (5%)	0 (0%)	
Sufentanil	12 (32%)	0 (0%)	
*Hypnotics*			0.3
Midazolam	31 (82%)	9 (100%)	
Propofol	7 (18%)	0 (0%)	
*Stay and extubation*			
Length of stay	9 [4, 19]	10 [4, 29]	0.3
Time interval between inclusion and extubation (days)	2 [0, 4] *n* = 35	1 [1, 11] *n* = 7	0.8
Extubation within 24 h	16 (46%)	4 (57%)	0.7
Extubation within 48 h	23 (66%)	4 (57%)	0.7
Time interval between inclusion and discharge from the PICU (days)	4 [2, 11]	6 [3, 17]	0.6
Time interval between extubation and discharge from the PICU (days)	3 [1, 7]	4 [2, 6]	0.6

PELOD2: Paediatric Logistic Organ Dysfunction 2, pSOFA: Paediatric Sequential Organ Failure Assessment, PIM III: Paediatric Index of Mortality III, MBP: mean blood pressure, VT: tidal volume, CRP: C-reactive protein; PICU: pediatric intensive care unit

### HRV and MODS

None of the HRV indices was correlated with the PELOD2 score. Energy was significantly but weakly correlated with the pSOFA score (r²=−0.31, *p* = 0.03). HFnu was significant correlated with the Phoenix score (r²=0.42, *p* = 0.003). (Table 3)

**Table 3:** Spearman’s coefficients for the correlations between HRV indices and the PELOD2, pSOFA, Phoenix scores and C-reactive protein

	PELOD2		pSOFA		Phoenix		C-reactive protein	
	*r*²	*p*-value	*r*²	*p*-value	*r*²	*p*-value	*r*²	*p*-value
Energy	−0.25	0.094	**−0.31**	**0.03**	−0.24	0.11	−0.34	0.079
SDNN	−0.22	0.13	−0.25	0.085	−0.16	0.3	−0.32	0.11
RMSSD	−0.19	0.19	−0.16	0.3	0.01	> 0.9	−0.24	0.22
ANI	−0.25	0.097	−0.12	0.4	0.16	0.3	−0.16	0.4
LF	−0.22	0.13	−0.26	0.082	−0.16	0.3	−0.31	0.12
HF	−0.21	0.16	−0.19	0.21	−0.04	0.8	−0.31	0.11
HFnu	−0.03	0.8	0.24	0.10	**0.42**	**0.003**	0.070	0.7

PELOD2: Paediatric Logistic Organ Dysfunction 2, pSOFA: Paediatric Sequential Organ Failure Assessment, SDNN: standard deviation of normal to normal, RMSSD: root mean square of successive differences between adjacent RR intervals, ANI: analgesia nociception index, LF: low frequency, HF: high frequency, HFnu: normalized high frequency

HFnu was significantly higher in the moderate/severe MODS group than in the no/mild MODS group (0.35 vs. 0.28, respectively; *p* = 0.019) and discriminated well between the moderate/severe MODS and no/mild MODS groups (AUROC = 0.75, Se = 0.78, Sp = 0.74). Energy was lower (albeit not significantly) in the moderate/severe MODS group (0.17, vs. 0.35 in the no/mild MODS group; *p* = 0.086) and discriminated well between the two groups (Se = 0.78) (Table 4).

**Table 4:** HRV characteristics and the AUROC for discriminating between the no/low MODS and medium/high MODS groups

	**No/mild MODS**, *N* = 38	**Moderate/severe MODS**, *N* = 9	*p*-value	AUROC	Sp	Se
Energy	0.35 (0.18, 0.65)	0.17 (0.16, 0.27)	0.086	0.69	0.61	0.78
SDNN	16 (8, 36)	9 (7, 11)	0.2	0.65	0.58	0.89
RMSSD	8 (4, 31)	9 (6, 12)	0.6	0.44	0.40	0.78
ANI	70 (59, 86)	72 (57, 95)	0.7	0.54	0.90	0.33
LF	0.08 (0.02, 0.46)	0.04 (0.02, 0.04)	0.2	0.63	0.55	0.89
HF	0.02 (0.01, 0.16)	0.02 (0.01, 0.03)	0.6	0.45	0.53	0.67
HFnu	0.28 (0.20, 0.32)	0.35 (0.32, 0.37)	0.019*	0.75	0.74	0.78

AUROC: area under the receiving operator curve, Sp: specificity, Se: sensitivity, SDNN: standard deviation of normal to normal, RMSSD: root mean square of successive differences between adjacent RR intervals, ANI: analgesia nociception index, LF: low frequency, HF: high frequency, HFnu: normalized high frequency

When we tested for correlations between the three scores, we found that the pSOFA score was strongly correlated with the PELOD2 score (r²=0.62, *p* < 0.001) and the Phoenix score (r²=0.86, *p* < 0.001). The PELOD2 score was strongly correlated with the Phoenix score (r² = 1, *p* < 0.001).

To determine whether or not the HFnu and Energy were associated with MODS, we compared the two after adjusting for FiO_2_, SpO_2_, FiO_2_/SpO_2_, sex, and catecholamine administration. The groups differed significantly with regard to HFnu, after adjustment for SpO_2_ and sex. In contrast, no significant between-group differences were found after adjustment for FiO_2_ and catecholamine administration. After adjustment, the groups did not differ significantly with regard to Energy (Table 5).

**Table 5:** OR [95% confidence interval] for HFnu and energy as discriminants for MODS

HRV index		Log OR [95%CI]	*p*-value
HFnu	Unadjusted	12 [2.0, 24]	0.031
	FiO_2_	8.6 [−1.7, 21]	0.12
	SpO_2_	13 [2.8, 26]	0.023
	SpO_2_/FiO_2_	8.4 [−1.4, 20]	0.12
	Sex	12 [1.6, 26]	0.041
	Catecholamines	10 [−1.3, 24]	0.10
Energy	Unadjusted	−3.2 [−8.5, −0.18]	0.13
	FiO_2_	−2.9 [−8.4, 0.29]	0.2
	SpO_2_	−2.8 [−8.1, −0.16]	0.2
	SpO_2_/FiO_2_	−3.7 [−10, −0.20]	0.14
	Sex	−2.8 [−8.0, 0.15]	0.2
	Catecholamines	−2.3 [−7.6, 0.40]	0.2

HRV: heart rate variability, HFnu: normalized high frequency

## Discussion

In the present study, Energy was the only HRV index found to be significantly (and inversely) correlated with the pSOFA score. HFnu was the only HRV index that differentiated very reliably between the two groups, with higher values in the moderate/severe MODS group. Energy was lower (albeit not significantly) in the moderate/severe MODS group. These results could be interpreted as reflecting a decrease in overall ANS activity and a relative increase in parasympathetic activity in the patients in the moderate/severe MODS group. The lack of between-group differences in the ANI and the COMFORT B scale confirmed the groups’ similarity with regard to analgesia and sedation. The only confounding factors to have influenced HFnu were catecholamine administration and FiO_2_.

Our previously published, systematic review of the literature on MODS showed that the overall HRV variability (SDNN), spectral power (HF and LF) and signal complexity tended to be lower in patients with severe MODS [7]. Most studies of the relationship between HRV indices and MODS showed that the SDNN and the LF/HF ratio were best able to discriminate between severe MODS and low MODS [16–21]. In the present study, SDNN, Energy, LF and HF tended to be lower in the moderate/severe MODS group, although the differences with the no/mild MODS groups were not significant. These non-significant results might be due to the small number of patients in the moderate/severe MODS group. Although the LF/HF ratio was not computed in our study, we evaluated the HFnu = HF/(HF + LF); it was significantly lower in the moderate/severe MODS group. These results are in line with the reports by Schmidt et al. (2005) [16] and Zhang et al. (2014) [21]. Indeed, Schmidt et al. (2005) demonstrated that SDNN, LF, HF and LF/HF were significantly lower in patients with MODS than in patients without MODS [16]. Similarly, Zhang et al. (2014) showed that LF and LF/HF were significantly lower in patients with MODS than in patients without MODS [21].

Although several other studies have evaluated the ability of overall HRV (SDNN) to assess MODS [16, 17, 21], the Energy variable (specific to the ANI monitor) has never been studied in the context of patient presenting with MODS in the ICU or the PICU. However, Aragon et al. has looked at whether Energy can differentiate between COVID-19 survivors and non-survivors. In fact, the mean ± standard deviation Energy value was significantly lower in the non-survivors (0.322 ± 0.351) than in the survivors (0.587 ± 0.432; *p* = 0.001). However, Energy was not correlated with the SOFA score [22].

Only Badke et al. (2021) have studied HRV and MODS in a pediatric population in the PICU [23]. However, the researchers studied new-onset or progressive MODS (NPMODS), rather than the absolute pSOFA value. The HRVD score was higher in patients with NPMODS or who died, and discriminated between the latter and patients without MODS (AUROC: 0.69 for patients with NPMODS and 0.80 for patients who died). Badke et al. also observed that the overall ANS variability was lower when MODS occurred. However, Badke et al.’s results are difficult to compare with ours because the two study populations differed with regard to the ventilation modalities, treatments, and catecholamine administration.

In this secondary analysis, we demonstrated a decrease in HFnu and a tendency toward a decrease in LF, with no change in HF. These results may seem counterintuitive. Indeed, in our recent review, we observed that MODS was associated with a decrease in overall variability (SDNN), a decrease in both LF and HF power [7]. Only Zhang et al. showed a decrease in LF and no change in HF as demonstrated in our study [21]. On the other hand, when looking in the balance between the parasympathetic and sympathetic systems (HFnu or LF/HF), our results (increase in HFnu) confirmed the data in the literature showing a decrease in LF/HF [16–18, 21] and an increase in HFnu [21].

In our study, HFnu displayed statistically significant differences between groups, while ANI did not. Though HFnu and ANI both represent the relative ANS parasympathetic activity, the way these two HRV indices are calculated differs, which may explain this difference in significance. Indeed, HFnu represents the normalized HF content defined as HFnu = HF/(HF + LF) and is therefore computed on the spectral domain using a wavelet transform. On the other hand, ANI use a wavelet transform to filter the signal in order to retain the HF content only and then compute the magnitude of the resulting oscillation in the time domain. The computational difference between HFnu and ANI may be the only explanation to the differences observed in this study.

Many of the reports on studies of HRV in ICU patients with MODS do not describe the patient inclusion criteria. Some reports specify the percentage of patients with ventilatory support, sedation, or catecholamines but did not take into account the potential bias that these variables could represent [16, 18, 20, 23, 24]. Indeed, a systematic review showed that the values of HRV variables change (particularly for LF, HF and LF/HF) when a patient is weaned off invasive mechanical ventilation [25]. In our systematic review of the literature on HRV and MODS, only one study adjusted its models for various confounding factors, such as mechanical ventilation, catecholamines, dexmedetomidine and albuterol; however, the study did not have a standardized population [23].

In contrast to the other studies in this field, our patient population was standardized in terms of age, sedation, and ventilation conditions. Even so, our results showed that catecholamine administration and ventilation both influence HRV indices.

Our study had several limitations. The ANI EP which provided the data for this secondary analysis was not pre-specified to meet the research objective. Secondly, the two groups of patients differed with regard to certain variables, and the number of patients in the moderate/severe MODS group was low. However, our results were still significant. Thirdly, our HRV analysis covered a short period (10 min), relative to the 24 h over which MODS scores are calculated. However, this 10-minute period is in line with the European Society of Cardiology and North American Society of Pacing and Electrophysiology Task Force’s guidelines on HRV analysis [14].

Though this 10-minute period was standardized and free of any care stimulation, other environmental stimuli like light, noise or the presence of parents or caregiver were not condoled. However, these phenomena only slightly influence, or do not influence at all, the patient in a situation of deep sedation. In this study, we only analyzed simple, well-known time-domain and spectral HRV variables and nonlinear indexes (e.g. the approximate entropy or the sample entropy) were not explored. The use of more complex mathematical algorithms would perhaps have led to better understanding. However, they express the complexity of the signal, and it is more complicated to link them to one or another branch of the ANS, making their physiopathological interpretation difficult.

Moreover, pSOFA, PELOD-2, and Phoenix scores were computed post hoc which can introduce bias due to incomplete, retrospectively extracted data and potential inaccuracies in timing or missing clinical variables. However, data completeness was verified and any missing data were considered normal as stated in the scores computation guidelines.

Finally, the small sample size and the imbalance between the groups limit the statistical power and robustness of the multivariate models. Consequently, despite attempts at adjustment, the small sample size does not allow for reliable control, and after adjustment, many associations lost their statistical significance, suggesting residual confounding factors (catecholamine administration, FiO_2_, inflammatory status…). These results therefore need to be confirmed in a larger and more standardized population in a prospective clinical study.

## Conclusion

The results of our study in a PICU showed that HRV indices can differentiate between deeply sedated patients with MODS and those without. Further investigations are needed to confirm this finding and extend it to other populations.

## Data Availability

No datasets were generated or analysed during the current study.
